# Malaria Epidemiological Characteristics in Urban and Suburban Areas of Bamako, Mali

**DOI:** 10.1155/japr/8652385

**Published:** 2026-06-11

**Authors:** Abdoulaye Kassoum Kone, Boubacar Maiga, Amagana Dolo, Bahiry Camara, Adama Traoré, Jean Douba Mounkoro, Sayon Kamissoko, Kassoum Kayentao, Mouctar Diallo

**Affiliations:** ^1^ Parasites and Microbes Research and Training Center, University of Sciences, Techniques and Technologies of Bamako, Bamako, Mali, usttb.edu.ml; ^2^ National Blood Transfusion Center, Bamako, Mali; ^3^ Confessional Health Center of Nafadji, Bamako, Mali; ^4^ Community Health Care Center (CScom) of Dravéla, Bamako, Mali

**Keywords:** Bamako, comorbidities, malaria, *P. falciparum*, suburban area, urban

## Abstract

**Introduction:**

Malaria remains a major health problem in sub‐Saharan Africa. Malaria burden may vary according to different settings in a city. We aim to describe malaria epidemiology in urban and suburban areas in Bamako, capital of Mali.

**Methods:**

A cross‐sectional study was conducted in children and adults seeking care with febrile illnesses in urban and suburban community health centers in Bamako. The Community Health Care Center (CScom) of Dravéla in urban setting and the Confessional Health Center of Nafadji in suburban setting were selected. In Dravéla, participants of all age categories with fever (or a history of fever) were enrolled. In Nafadji, children of 0–59 months old, with fever (or a history of fever in the last 24 h), were enrolled. In addition to clinical signs and parasitological, sociodemographic parameters were also assessed.

**Results:**

*Plasmodium falciparum* malaria prevalence was, respectively, 68% and 61.16% in Dravéla and Nafadji. Severe malaria cases have been reported in Nafadji, including cerebral malaria (15.15%) and severe malaria anemia (1.64%). Self‐medication and use of impregnated mosquito nets were, respectively, 45.5% and 69.5% in Dravéla, and 32.8% and 39.8% in Nafadji.

**Conclusion:**

Our results suggest that malaria is the main cause of fever and morbidity in peri‐urban and urban areas of Bamako. The impregnated mosquito nets were less frequently used in the suburban area. In Bamako, during the dry season, malaria transmission is high; control efforts and tools should be maintained during that period.

## 1. Introduction

Malaria, a major parasitic endemic disease, is still a public health problem in sub‐Saharan Africa. Among parasite species infecting human, *Plasmodium falciparum* causes most of malaria morbidity and mortality. Africa remains the area most affected by malaria in the world, with 90% of deaths; 75% of the lethality occurs in children under 5 years old [1]. In Mali, as in the majority of sub‐Saharan countries, malaria remains the major endemic parasitic disease and the leading cause of morbidity and mortality among the most vulnerable groups, especially children under 5 and pregnant women [2]. Cerebral malaria (CM) and severe anemia are the common severe forms in the urban area [3, 4]. The most common clinical presentations among children with severe malaria were fever 722 (97.3%), cough 478 (64.2%), and difficulty in breathing 122 (17.9%) in Uganda [5].

Human behaviors, such as the use of long‐lasting insecticide nets (LLINs), provided no protection against malaria in urban and peri‐urban settings [6]. Patients and health care workers′ behaviors and attitudes, along with limited access to health care centers, are the main challenges for health authorities in fighting malaria in endemic countries. These behavioral factors and limited access to health care services may be linked to urbanization styles. A better understanding of the malaria characteristics for each residential area could help to better adapt prevention measures and awareness‐raising strategies for a favorable behavioral change. Urbanization has an impact on public health. The increase in the rate of urbanization, combined with the reduction of rural population, will lead to a shift in disease morbidity patterns. The pattern, where infectious diseases are more frequent in rural areas while noncommunicable diseases are more frequent in urban areas, could be modified [7]. The rate of urbanization is now very high in sub‐Saharan Africa. It is estimated that 75% of the population in South Saharan Africa could be urbanized by 2050 [8].

In 2019, Mali was 41% urbanized, and it is expected to cross the 48% mark by 2030. The rapid and anarchic development of urban areas increases the risk for the development of mosquito breeding sites and malaria transmission. Given the speed of urbanization of the city of Bamako, there is a need for adapted malaria control strategy. We aimed to determine the sociodemographic and behavioral characteristics associated to malaria morbidity in urban and suburban areas of Bamako.

## 2. Materials and Methods

### 2.1. Study Sites

**Figure 1:**
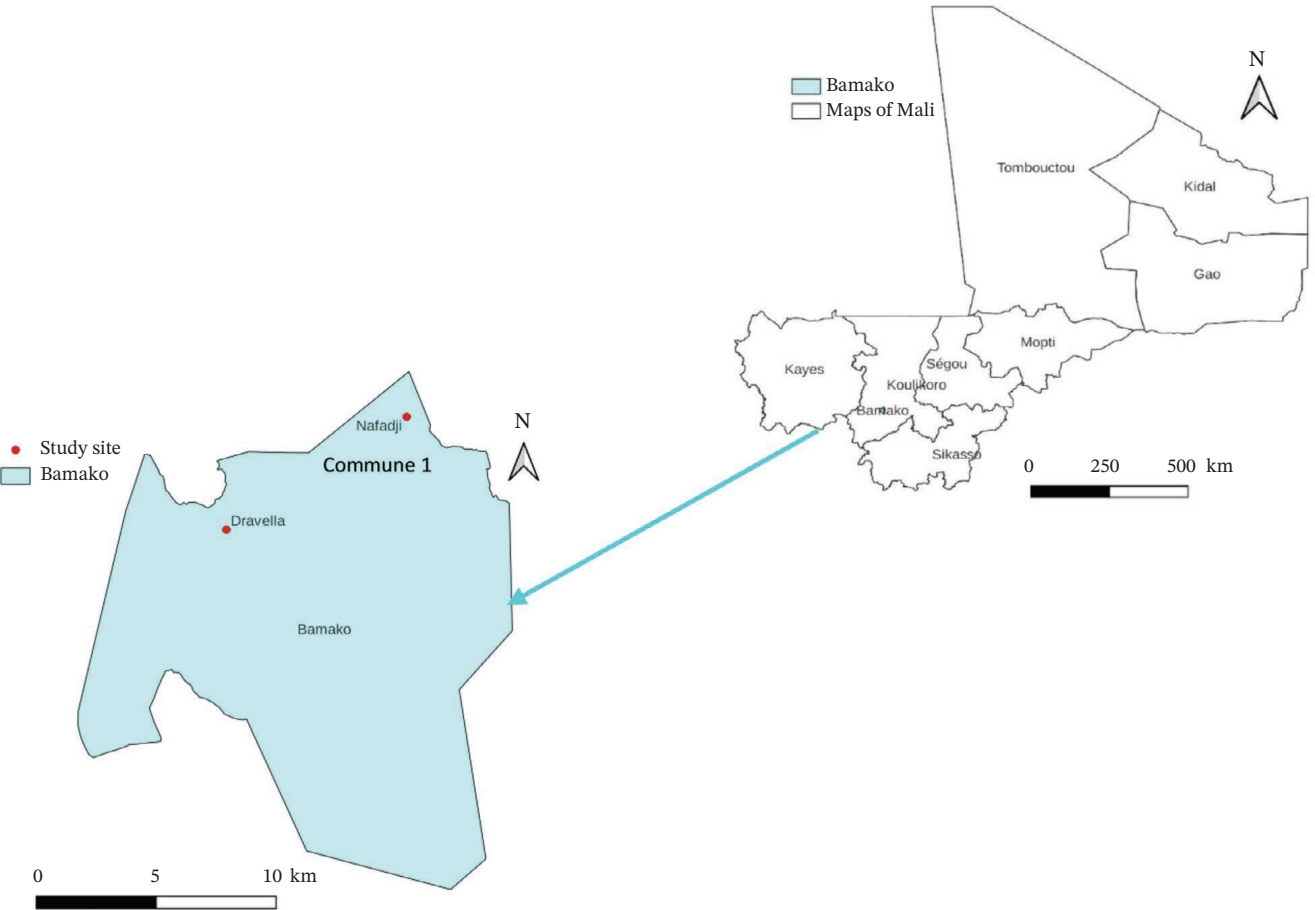
Map of Mali, with location of the district of Bamako and study sites.

The study was carried out in two community health care centers (CScom) named ASACODRAB and Catholic Health Center of Nafadji “Paroisse Saints Martyrs de l′Ouganda,” both located in Bamako district, the capital city of Mali. ASACODRAB is located in Dravéla quarter, in an urban area in Commune III. It was built during the colonial period in 1940 to provide free care to the population of the downtown of Bamako city. The Catholic Health Center of Nafadji, recently erected, is located in Nafadji quarter, in the suburban area of Commune I of the district of Bamako (Figure 1).

### 2.2. Study Period

The study was conducted from September to December 2015 (4 months) in Dravéla, corresponding to the end of the malaria transmission season in Mali and the beginning of the cold dry season. In Nafadji, data were collected from January to June 2017 (6 months), corresponding to the dry season with low malaria transmission.

### 2.3. Type of Study

A cross‐sectional survey was conducted in both sites. The study participants′ sociodemographic, epidemiological, clinical, and parasitological data were collected.

### 2.4. Study Population and Sampling

The study population concerned all age groups and gender seeking care in ASACODRAB. It was limited to children aged 0–5 years seeking care in the Catholic Health Center of Nafadji.

### 2.5. Inclusion Criteria and Exclusion Criteria

#### 2.5.1. Inclusion Criteria

In the ASACODRAB urban area, any patients presenting fever or a history of fever 72 h before admission and consenting to participate in the study were enrolled.

At the Catholic Health Center of Nafadji, suburban area, children aged 0–59 months old, who were feverish on admission (temperature ≥ 38°C) or had a history of fever for 24 h, and whose parents consented and agreed to answer the questionnaire were enrolled.

#### 2.5.2. Exclusion Criteria

In Dravéla and Nafadji, patients known to be carriers of chronic conditions, such as tuberculosis, HIV/AIDS, diabetes, and hypertension, were not enrolled.

### 2.6. Sample Size Calculation and Sampling

In Dravéla, based on a malaria prevalence of 40%, an accuracy of 5%, 10% of unusable records or missing data, and a confidence interval between 35% and 45%, we estimated the sample size of 406 participants.

In Nafadji, sampling was exhaustive; all patients who fulfilled the inclusion criteria were enrolled.

### 2.7. Study Procedures

At enrollment, sociodemographic and behavioral data were collected using survey forms by interviewing adult participants or children′s parents or tutors. The clinical presentation data were obtained by interview, a clinical examination was performed, and body temperature was recorded.

#### 2.7.1. Malaria and Other Disease Diagnosis

In Dravéla and Nafadji, malaria was diagnosed using microscopy. The thick smears were dried at room temperature away from dust and flies, then stained with 3% GIEMSA diluted in buffered water for 40 min. They were then rinsed and dried before reading. The SD BIOLINE Malaria Antigen p.f. test that contains a strip coated with a mouse monoclonal antibody specific to Histidine‐Rich Protein II (HRP‐II) was used.

In Davela, typhoid and paratyphoid diagnosis was done using the Widal–Felix test. In Nafadji, other additional tests if necessary were used.

The participants with negative malaria smears received an appropriate treatment for the other diseases diagnosed.

#### 2.7.2. Definition of Malaria Clinical Attacks

Severe malaria was defined by the presence of a WHO severe malaria criteria associated with the presence of trophozoites of *P. falciparum* [9], whereas uncomplicated malaria was defined by the presence of clinical signs (body temperature ≥ 38°C or a history of fever, chills, headaches, and digestive disorders for 24 h) associated with the presence of *P. falciparum* trophozoites and/or a positive Rapid Diagnostic Test (RDT).

#### 2.7.3. Study Variables

The variables assessed were age, gender, weight, occupation, use of impregnated or untreated mosquito nets, self‐medication, spleen size, body temperature, clinical signs, malaria episodes, nonfebrile diseases, and malaria thick smear results (positive or negative).

### 2.8. Data Analysis

The data were entered into Access 2007 and Excel 2007 and then exported to SPSS Version 20 or STATA for statistical analysis. Chi‐square or Fisher tests were used; *p* value ≤ 0.05 was considered statistically significant.

### 2.9. Ethical Aspects

The community and health authorities′ permission was obtained before the study initiation. Study purpose and procedures were explained to participants or the children′s parents and guardians [10].

## 3. Results

### 3.1. Overall Results

In the Nafadji health care center, from January to June 2017, 3104 children (aged 0–14 years) sought care, of whom 1885 were aged 0–5 years old (60.73%). Out of those, 632 had a fever or a history of fever in the 24 h prior seeking care (33.53%), children age group 12–36 months represented 60.85% (185/304), and parasitemia class 1–1000 trophozoites/*μ*L was common, observed in 88.16% (268/304).

In Dravéla, patients from outside the ASACODRAB health area were predominant (66%). Feverish patients were the most represented (80%). Parasitemia class ≥ 10,001 trophozoites/*μ*L was more common (48.3%). The age group older than 15 years was the most represented (60.4%).

In both sites, *P. falciparum* was the only species identified.

### 3.2. Sociodemographic Characteristics

Distribution of patients by age in the two sites: A total 505 participants were included in Dravéla, among whom patients older than 15 years were predominant 305/505 (60.4%), followed by children aged 0–5 years old (18.4%). In Nafadji, a total of 632 participants aged from 0 to 5 years old were enrolled.

Self‐medication and use of impregnated mosquito nets: The frequency of self‐medication was, respectively, 45.5% and 32.8% in Dravéla and Nafadji (Table 1). The frequency of regular use of impregnated mosquito nets was, respectively, 69.5% and 39.80% in the urban area and Nafadji (Table 1).

**Table 1:** Practical attitudes and behavior of patients in Dravéla and parents in Nafadji.

**Dravéla**	**Use of medication before admission (*n*=505)**	**Effective**	**%**
	Self‐medication	230	45.5
	Absence of self‐medication	275	54.5
	**Use of mosquito nets (*n* = 505)**	**Effective**	**Yes (%)**
	Regular impregnated	154	351 (69.5)
	Irregular impregnated	454	30 (6.0)
	Not impregnated	497	8 (1.6)

**Nafadji**	**Use of medication before admission (*n* = 304)**	**Effective**	**%**
	Self‐medication	100	32.89
	Absence of self‐medication	204	67.11
	**How antimalarials were administrated (*n*=100)**	**Effective**	**%**
	Health worker	33	33
	Traditional medicine	28	28
	Self‐medication	39	39
	**Use of impregnated mosquito nets (*n* = 304)**	**Effective**	**%**
	Regular	121	39.8
	Irregular	135	44.4
	Do not use	48	15.7

### 3.3. Clinical Presentations

**Figure 2:**
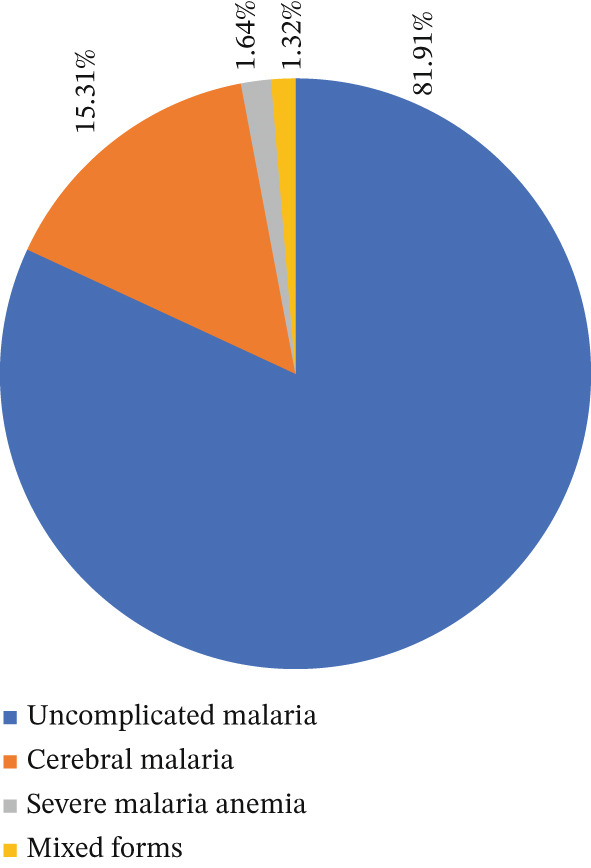
Malaria clinical presentations distribution in Nafadji Health Care Center.

**Figure 3:**
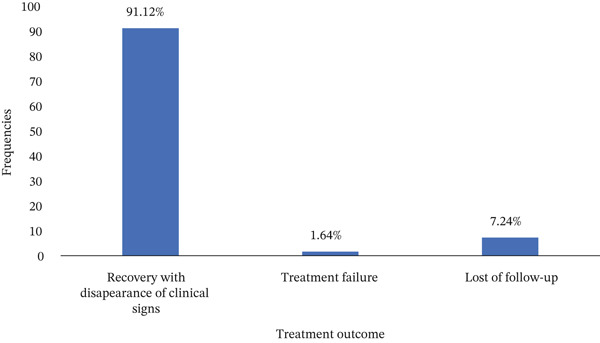
Clinical evolution of participants in Nafadji Health Care Center.

The most frequent clinical sign in the two sites was history of fever (77.45%) in Dravéla, fever (body temperature ≥ 38°C, 100%) in Nafadji, followed by digestive signs and respiratory signs (Table 2). Cases of uncomplicated malaria were identified in Dravéla; however, severe malaria and uncomplicated malaria cases were recorded in Nafadji (Figure 2). In Nafadji, 81.91% were uncomplicated malaria cases, 15.15% were CM, and 1.64% were severe malaria anemia (SMA) (Figure 2). The post treatment recovery rate, the loss of follow‐up rate, and treatment failure rate were, respectively, 91.12%, 7.24%, and 1.64% (Figure 3).

**Table 2:** Frequency of clinical manifestations at admission at the two sites.

Sites	Clinical manifestations	Effective	%
**Dravéla (n=479)**	Fever	371	77.45
	Nausea	205	42.58
	Vomiting	168	35.07
	Diarrhea	47	9.8
	Cough and rhinorrhea	144	30.06
	Headaches	333	69.51
	Aches	346	72.23
	Anorexia	421	87.89
	Dizziness	210	43.84
	Asthenia	378	78.91

**Nafadji (n=304)**	Fever	304	100
	Digestive signs	285	93.75
	Cough and rhinorrhea	133	43.75
	Seizures	18	5.92
	Headaches	36	11.84
	Prostration	43	14.14
	Respiratory distress	03	0.99
	Chills	19	6.25
	Jaundice	8	2.63
	Pallor	24	7.89

Malaria was the leading cause of morbidity in the both centers with frequencies of 68% in Dravéla and 61.16% in Nafadji. Malaria is followed by respiratory infections (22.13%) (Table 3).

**Table 3:** Frequencies of malaria and other febrile illnesses in the two health centers.

Febrile illnesses	Dravéla		Nafadji	
	Effective	%	Effective	%
Malaria	343	68	304	61.16
Respiratory tract infections	68	13.5	110	22.13
Typhoid fever	52	10.3	8	1.6
Pharyngitis	30	6	—	—
Gastroenteritis	—	—	42	8.45
Others (parotitis, allergic reactions, gastroenteritis)	12	2.4	11	2.21
Total	505	100	497	100

### 3.4. Parasitological Findings

**Figure 4:**
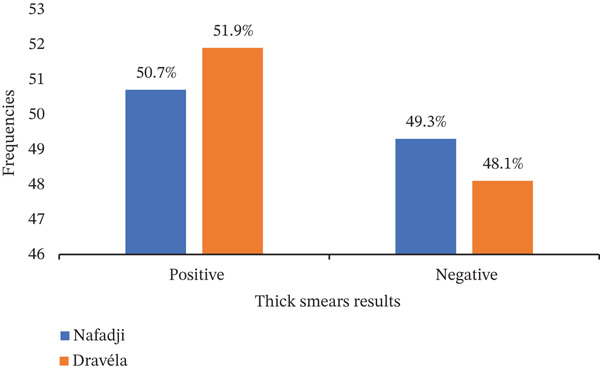
Results of microscopic examination of thick smears in children aged 0–5 years.

Microscopic examination of the thick smears had shown overall positivity rates of 71% and 48.10%, respectively, in Dravéla and Nafadji centers (Table 4). In children aged 0–5 years, the proportion of positive malaria smear observed was, respectively, 50.70% and 48.10% in Dravéla and Nafadji (Figure 4).

**Table 4:** Results of microscopic examination of thick smears in patients with suspected malaria.

Thick smears	Dravéla		Nafadji		*p*
	Effective	%	Effective	%	
Positive	328	71	304	48.10	0.001
Negative	134	29	328	51.90	
Total	462	100	632	100	

## 4. Discussion

The study examines malaria morbidity, self‐medication, and the use of insecticide‐treated mosquito nets. The clinical malaria clinical features in the urban and suburban areas of Bamako have shown similar malaria prevalence; however, different methodological approaches between the urban and the peri‐urban areas were used. In Dravéla, an urban area, the study included participants of all age categories, whereas in Nafadji, a peri‐urban area, it focused on children from 0 to 5 years old, in whom a systematic body axillary temperature measurement was performed. All febrile cases and/or a history of fever 24 h before admission went through a full clinical examination and parasitological assessment. In Dravéla, the survey was conducted at the end of the malaria transmission season, from September to December, whereas in Nafadji, it was carried out in the dry season before the malaria transmission season from January to June. Both studies were carried out outside the period of intense malaria transmission: from September to December 2015 in urban areas (4 months) and for the suburban area CScom from January to June 2017 (6 months). Differences in the methodological approach between Dravéla and Nafadji highlighted the burden of malaria in the urban and suburban areas during the dry season in Bamako. The malaria transmission pattern may explain the difference in morbidity observed between the two study sites.

We observed that self‐medication was more prevalent in the urban area than in the peri‐urban area. This attitude could lead to misdiagnosis [11], with a malaria clinical episode being misdiagnosed at admission, such as presentation of asymptomatic and nonfebrile patients with microscopic or submicroscopic parasitemia. The consequences are a slow evolution to severe malaria. Several studies′ findings have shown that self‐medication with antimalaria drugs is common in sub‐Saharan African. A community‐based cross‐sectional survey conducted in Kakumiro District in August 2023, in Uganda, has shown a prevalence of 62% (95% CI 58.2–65.9%) self‐medication for malaria [12], and in Nigeria women practice self‐medication with antimalarials both for themselves (42.7%) and for their newborn children (22.7%) [13].

Despite the difference in transmission season and age groups, the frequency of regular use of insecticides‐treated mosquito nets as a preventive measure against malaria was pretty good in urban areas (69.5%) where the inhabitants are better educated and richer than in the suburban area (39.80%) (*p* < 0.001). Elsewhere, a frequency of LLIN usage of 22.2% in the suburban area in Ouagadougou in Burkina Faso [14] was lower than the rate we observed during the current study. Similar LLIN usage was reported in Ethiopia [15]. In Malawi, no association was found between the use of LLINs and protection from malaria in urban and peri‐urban areas [6].

The most frequent clinical signs were fever and digestive disorders signs. Malaria has been the leading cause of morbidity in both health centers although the study periods were different during the current study. In the internal medicine department of a teaching hospital in Bamako, fever was reported in 28.8% of patients, and malaria was the second cause of morbidity after respiratory diseases [16]. These differences in clinical signs profile between our study and others could be due to study design, the study periods, and health care setting types.

Uncomplicated malaria was predominant in the two centers. However, we observed 15.15% of severe malaria in Nafadji, and no cases of severe malaria were recorded in Dravéla. This could be explained by the fact that patients with signs of severe malaria are referred directly to the Reference Health Center of Commune III or to the teaching hospital Gabriel Touré, near the Dravéla CSCom, where treatment of severe malaria was available and provided in accordance with the NMCP. In Nafadji, 81.91% of cases were uncomplicated malaria cases, 15.15% were CM, and 1.64% SMA. CM was more frequent than SMA. Similar tendency distribution of CM and SMA has been found by Ranque et al. (2008) on a study performed exclusively on severe malaria at the pediatric ward of Gabriel Toure Hospital in Bamako in children during the malaria transmission season from 1999 to 2002. Despite the difference in study design, they found 66% of CM and 34% of SMA [3].

We found that malaria prevalence was high in urban and semiurban areas, and the malaria prevalence was even higher in the urban area in Dravéla (71.0%) than in the suburban area in Nafadji (48.1%). This difference could be explained by the study period and population, and/or the heterogeneity of the malaria transmission in urban areas with an adaptation of the mosquito genus *Anopheles* in human‐made breeding sites [17]. Thus, our findings confirm the concept of pattern change with the urbanization. In Bamako, in November, Doumbo et al. [18] did not observe any statistically significant relationship between urban and suburban areas (*p* > 0.05) regarding the *Plasmodium* spp. index in children. We observe a malaria prevalence of 61.16% in the peri‐urban area; similarly in Benin, malaria prevalence was high (68.9%) in the peri‐urban area on the coast, quite higher than our finding [19]. The malaria transmission is more intense in the suburban area where larval breeding sites are conducive to the development of *Anopheles* spp. than in urban areas where the ecosystem is not favorable to the development of *Anopheles* spp. [18].

In our study, *P. falciparum* was the only species identified at microscopy; the prevalence of positive malaria smear among children aged 0–5 years was comparable between Dravéla and Nafadji (50.7% vs. 48.1%). A facility‐based cross‐sectional study conducted in six health facilities in the Nandom Municipality in July 2024 in Ghana reported a prevalence 26.0% (CI 21.9–30.5) for microscopy [20] lower than that we reported; however, a microscopy positivity rate of 45.8% among febrile cases was found in Tanzania [21].

Postmalaria treatment failure and loss of follow‐up were, respectively, 1.64% and 7.24% in Nafadji; elsewhere, a 98% adherence to malaria treatment with fewer cases of treatment failure has been reported [21].

In recent years, several studies have been conducted on urban malaria using data from the literature review and a model approach on urban malaria studying the urbanization in sub‐Saharan Africa and implication for malaria control. The authors proposed an integrated approach to urban malaria control strategies including environmental management, vector control, and accurate diagnosis and prompt treatment of patients. [22]. Da Silva et al. [23] have studied the factors contributing to malaria transmission in urban settings; they found that the vectors adapt to the urban environment and the transmission is continuing, hence the importance of continuous monitoring and control in sub‐Saharan Africa. Another trend was found on the impact of urbanization on malaria in Senegal by Machault et al. [24]. The authors evaluated environmental data extracted from satellite images associated with *An. arabiensis* in urban Dakar and generated malaria transmission risk maps. Their findings indicated that there are benefits from urbanization in Dakar, as the proportion of the population at low risk increased as urbanization increased.

The weakness of our study is the lack of entomological data at the same period in Bamako. However, a study carried out in the urban area of Bamako in August and October in 2011 has shown that mosquitoes of the genus *Anopheles* are present and contribute to the transmission of malaria in foci of Bamako [25].

## 5. Conclusions

Our findings suggest that malaria burden is high in urban or suburban areas and showed that malaria transmission persists in the dry period in urban settings. Sensitization in the suburban area as well in the urban area is required to improve the use of insecticide‐treated mosquito nets. An entomological study during the dry and rainy seasons in urban and peri‐urban areas should be implemented for vector control.

## Author Contributions

**Boubacar Maiga:** conceptualization, methodology, resources, supervision, data curation, validation, writing – original draft preparation. **Sayon Kamissoko:** conceptualization, methodology, resources, supervision, data curation, validation. **Bahiry Camara:** conceptualization, methodology, resources, supervision, data curation, validation. **Adama Traoré:** investigation, data curation. **Jean Douba Mounkoro:** investigation, data curation. **Amagana Dolo:** data curation, writing – review & editing. **Abdoulaye Kassoum Kone:** validation, writing – original draft preparation. **Kassoum Kayentao:** validation, formal analysis. **Mouctar Diallo:** writing – review & editing.

## Funding

No funding was received for this manuscript.

## Disclosure

All authors read and approved to the published version of the manuscript.

## Conflicts of Interest

The authors declare no conflicts of interest.

## Data Availability

The data that support the findings of this study are available from the corresponding author upon reasonable request.
